# An open-source, MATLAB based annotation tool for virtual slides

**DOI:** 10.1186/1746-1596-8-S1-S30

**Published:** 2013-09-30

**Authors:** Riku Turkki, Margarita Walliander, Ville Ojansivu, Nina Linder, Mikael Lundin, Johan Lundin

**Affiliations:** 1 Institute for Molecular Medicine Finland (FIMM), P.O. Box 20, FI-00014 University of Helsinki, Helsinki, Finland; 2 Center for Machine Vision Research, Department of Computer Science and Engineering, P.O Box 4500, FI-90014 University of Oulu, Finland; 3 Division of Global Health, Department of Public Sciences, Karolinska Institutet, Stockholm, Sweden

## Background

Computer aided analysis of virtual slide images has become an increasingly popular research topic and area of development. Novel applications are aimed for instance at automation of certain stages in sample assessment and assay readout (e.g. segmentation, detection, etc.) [2] or for reproducible measurement of a sample’s visual appearance (e.g. grading, morphological classes etc.) [3].

Statistical learning methods are one of the key algorithms used in building these image analysis applications. Especially supervised algorithms (e.g. support vector machines (SVM), AdaBoost, k nearest neighbour (kNN), etc.) offer a way to train a classifier that can perform complex quantification tasks. The process requires annotation of labelled examples in order to describe the task and learn a model. Recently, image acquisition techniques for large-scale digitization of tissue samples have become common and require new methods to perform the annotation and associated labelling [4].

In this study we present an annotation tool that combines 1) direct interaction with a remote slide collection (a web based virtual microscopy application), 2) an interface to annotate points of interest in the virtual slides and 3) fast transition from annotation to development of image analysis methods.

## Methods

The annotation tool is written in MATLAB (matrix laboratory) that is a cross-platform numerical computing environment (MathWorks, Natick, MA). MATLAB is a tool with a wide range of application areas and adopted by the image analysis community because of its versatile features. The programming language and its computing environment offers tools for testing and implementation of methods from a number manipulation to creating interactive graphical user interface (GUI) applications.

The annotation tool is based on exploiting two important properties of the current platform: 1) a *random code-stream access* featured image format (e.g. ECW or JPEG 2000) and 2) a *compression streamlining* protocol. The random code-stream compression makes it possible to extract sub-images from a large virtual slide file. Thus, the user can download only a field of interest, instead of loading the whole image file, which might be of gigapixel size. The other required property, the compression streamlining protocol, basically implements this sub-image extraction over standard hypertext transfer protocol (HTTP) in an efficient way.

**Figure 1:**
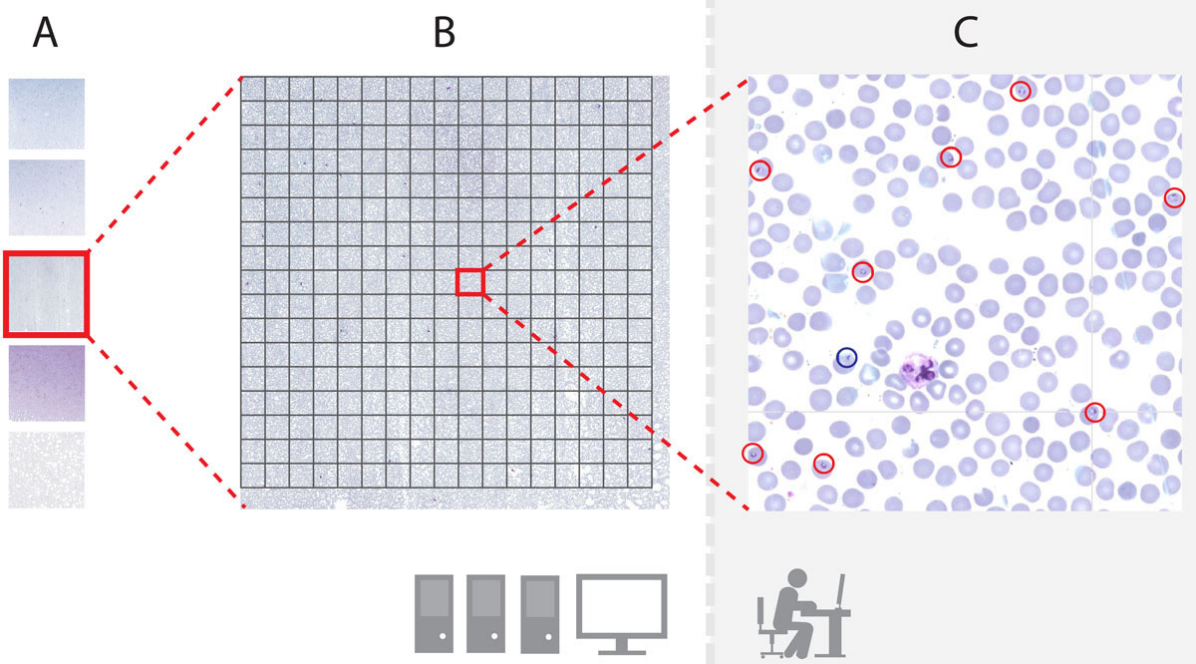
**The operational principal of the annotation tool** A) A slide collection saved at a remote virtual microscopy server, B) the tool reads the metadata of the slide and determines the coordinates for the tiles, C) a tile is downloaded and displayed to an annotator and the coordinate-label pairs are saved to a client computer.

With the advantages of the above-mentioned properties that a slide collection should have, it is reasonably straightforward to implement a MATLAB based tool to operate a remote database. The overall structure of the tool is illustrated in Figure 1. In a first phase the tool reads the metadata of a virtual slide file and deduces its height and width. Using a server’s streamlining protocol, uniform resource locators (URL) are defined to extract the tiles from the pre-calculated coordinates within the virtual slide. In the annotation phase, the tiles are extracted and downloaded one at the time from the server and displayed to an annotator and requested for annotations. The locations and labels of the annotations are saved locally in MAT files.

## Results and discussion

We implemented a MATLAB based annotation program capable of accessing a remote slide collection. The implementation is demonstrated within a previously described virtual microscopy environment [1,5] running image web server software (Erdas Inc., Atlanta Georgia), but is modifiable to pair with other platforms as well. The current virtual microscopy platform has proprietary server software (Image Web Server) that accepts both JPEG 2000 and ECW compressed files. The slides are made accessible via an *ImageX* protocol, which is implemented by the server. Other platforms, which have the ability to use JPEG 2000 files only, can utilize the JPIP (JPEG 2000 Interactive Protocol) to extract the metadata and tiles from a virtual slide over HTTP [6]. The code for the proposed tool is freely available [7].

The annotation tool keeps a record of all markings and enables the slides to be annotated in parts: it is possible to continue the annotation process from the beginning of a slide, or alternatively from a tile where the annotation was previously interrupted. The tool also keeps record of tiles that are already displayed to an annotator, which allows the areas that have not been annotated to be excluded from later processing if wanted. All the data are saved locally on the user’s computer in standard MAT files.

**Figure 2:**
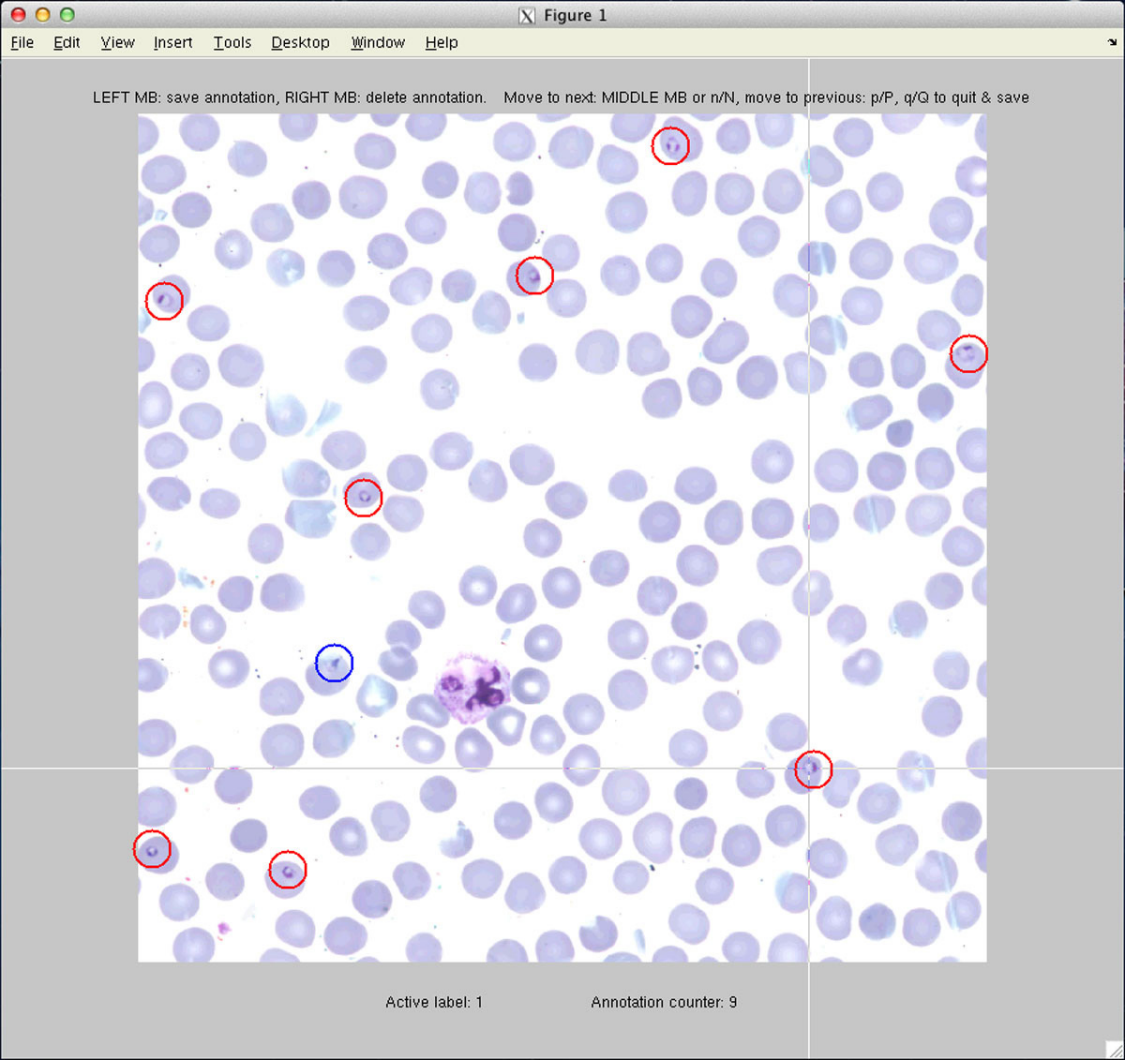
**The annotation window** Marked annotations are visualized with coloured circles to distinguish their labels. User can interact with the tool by using the mouse and the keyboard. The allowed user commands are displayed above the image.

The tiles are loaded one at the time from the image server and displayed to the annotator. The annotation window of an annotation process in a digitized thin blood smear film is illustrated in Figure 2. All the annotations are visualized with circles, which are coloured according to their labels. The tool can be set to handle at the most ten different labels.

The actual annotation process is kept as simple as possible to provide a fast and easy-to-use interface. The user is able to add and remove an annotation by simple mouse clicks: the left button for adding a new annotation and the right button to delete the nearest detected annotation. The keyboard’s number keys are used to set the active category label and by keys N (next) and P (previous) the user can move between the tiles.

The work was motivated by our own need to annotate large virtual slide collections. In addition to the proposed applications, the tool can be applied to any kind of analysis of virtual slides. For instance, the tool offers a way to compare the agreement and accuracy of human annotators to recognize and locate objects, or it can be used to deliver one’s annotations to a shared resource.

## Conclusions

We have described a simple and effective way to combine a computational environment with a virtual microscopy environment [1] to allow fast transition and iteration between method development and data annotation. The code for the annotation tool is freely available [7] and can be modified to suit different server settings. The use of the tool is demonstrated on a virtual microscopy platform by providing example slides of thin blood films.

## List of abbreviations

ECW: Enhanced Compressed Wavelet; GUI: Graphical User Interface; HTTP: Hypertext Transfer Protocol; kNN: k-Nearest Neighbour; SVM: Support Vector Machine; URL: Uniform Resource Locator

## Competing interests

The authors declare that they have no competing interests.

## Authors' contributions

RT planed and wrote the code for the annotation tool. MW and VO reviewed the implementations and suggested modifications. ML consulted on the use of the virtual microscopy platform. RT drafted the article and NL and JL contributed by supervision of the work and review of the manuscript. All authors read and approved the final manuscript.
